# Correlation of Somatostatin Receptor-2 Expression with Gallium-68-DOTA-TATE Uptake in Neuroblastoma Xenograft Models

**DOI:** 10.1155/2017/9481276

**Published:** 2017-08-08

**Authors:** Libo Zhang, Douglass C. Vines, Deborah A. Scollard, Trevor McKee, Teesha Komal, Milan Ganguly, Trevor Do, Bing Wu, Natasha Alexander, Reza Vali, Amer Shammas, Travis Besanger, Sylvain Baruchel

**Affiliations:** ^1^The Hospital for Sick Children, Toronto, ON, Canada; ^2^The STTARR Innovation Centre, University Health Network, Toronto, ON, Canada; ^3^The Center for Probe Development and Commercialization, Hamilton, ON, Canada

## Abstract

Peptide-receptor imaging and therapy with radiolabeled somatostatin analogs such as ^68^Ga-DOTA-TATE and ^177^Lu-DOTA-TATE have become an effective treatment option for SSTR-positive neuroendocrine tumors. The purpose of this study was to evaluate the correlation of somatostatin receptor-2 (SSTR2) expression with ^68^Ga-DOTA-TATE uptake and ^177^Lu-DOTA-TATE therapy in neuroblastoma (NB) xenograft models. We demonstrated variable SSTR2 expression profiles in eight NB cell lines. From micro-PET imaging and autoradiography, a higher uptake of ^68^Ga-DOTA-TATE was observed in SSTR2 high-expressing NB xenografts (CHLA-15) compared to SSTR2 low-expressing NB xenografts (SK-N-BE(2)). Combined autoradiography-immunohistochemistry revealed histological colocalization of SSTR2 and ^68^Ga-DOTA-TATE uptake in CHLA-15 tumors. With a low dose of ^177^Lu-DOTA-TATE (20 MBq/animal), tumor growth inhibition was achieved in the CHLA-15 high SSTR2 expressing xenograft model. Although,* in vitro*, NB cells showed variable expression levels of norepinephrine transporter (NET), a molecular target for ^131^I-MIBG therapy, low ^123^I-MIBG uptake was observed in all selected NB xenografts. In conclusion, SSTR2 expression levels are associated with ^68^Ga-DOTA-TATE uptake and antitumor efficacy of ^177^Lu-DOTA-TATE. ^68^Ga-DOTA-TATE PET is superior to ^123^I-MIBG SPECT imaging in detecting NB tumors in our model. Radiolabeled DOTA-TATE can be used as an agent for NB tumor imaging to potentially discriminate tumors eligible for ^177^Lu-DOTA-TATE therapy.

## 1. Introduction

Neuroblastoma (NB) is the most common extracranial childhood malignancy, responsible for 15% of all childhood cancer deaths [1]. Despite intensive treatment protocols including multimodal therapy with hematopoietic stem cell transplantation and immunotherapy, three-year disease-free survival is only about 60% for metastatic disease compared to 95% for localized tumors [2, 3].

Somatostatin receptors (SSTRs) are expressed at relatively low levels in most organs. They are moderately expressed in the brain, gastrointestinal tract, pancreas, kidney, and spleen. In contrast, SSTRs, especially SSTR2, have been shown to be highly expressed in various human tumors including pancreatic, small cell lung, and carcinoid tumors, as well as paraganglioma, pheochromocytoma, and neuroblastoma [4]. Georgantzi et al. demonstrated variable frequencies of somatostatin receptor (SSTR1-5) expression in 5 NB cell lines and 11 NB patient tumor biopsy samples [5], making molecular imaging and radionuclide therapy with somatostatin-based nuclear probes an attractive therapeutic option in appropriately selected patient populations [6].

DOTA-TATE is the somatostatin (cyclic peptide hormone) analog of Tyr3-octreotate (TATE) coupled with the macrocyclic chelator 1,4,7,10-tetraazacyclododecane,1,4,7,10-tetraacetic acid (DOTA). DOTA-TATE is SSTR2 selective with a higher SSTR2 affinity (~0.2 nM)* in vitro* comparing to two other commonly used somatostatin analogs [^68^Ga-DOTA^0^-Tyr^3^]octreotide (DOTA-TOC) and [DOTA^0^,1NaI^3^]octreotide (DOTA-NOC) [7]. Peptide-receptor imaging and therapy with radiolabeled somatostatin analogs are an established and effective treatment option for adult patients with SSTR-positive neuroendocrine tumors [4]. DOTA-TOC and DOTA-TATE can also be radiolabeled with ^90^Y or ^177^Lu for targeted *β*^−^-particle radionuclide therapy of neuroendocrine tumors. In a recent phase I trial, the safety and efficacy of ^90^Y-DOTA-TOC therapy were demonstrated in 17 children and young adults with refractory SSTR-positive neuroendocrine tumors including NB [8]. The first study of ^177^Lu-DOTA-TATE treatment in 35 patients with gastroenteropancreatic neuroendocrine tumors was published in 2003 where an objective response of 38% was achieved [9]. In a 2008 evaluation of 310 adult patients with neuroendocrine tumors, an overall response of 30% was reported [10]. More recently, a pilot clinical study demonstrated that ^68^Ga-DOTA-TATE PET could be used to image children with relapsed or primary refractory high-risk NB, and ^68^Ga-DOTA-TATE PET could be used to identify potential candidates for ^177^Lu-DOTA-TATE treatment [11]. In this study, 6 out of 8 children demonstrated high uptake of ^68^Ga-DOTA-TATE and proceeded to treatment. Patients received 2 or 3 administrations of ^177^Lu-DOTA-TATE (0.3 GBq/kg; 8.1 mCi/kg per dose) at a median interval of 9 weeks and a median administered activity of 7.3 GBq. Five of these patients had the stable disease as assessed using the Response Evaluation Criteria in Solid Tumors (RECIST). This study, while limited in the number of patients studied, provided proof-of-principle that children with NB can be imaged and treated with somatostatin receptor-targeted agents. More interestingly, this study demonstrated that 1 patient (out of a series of 6 patients) whose disease was negative for ^123^I-MIBG nevertheless demonstrated marked uptake of ^68^Ga-DOTA-TATE.

The primary purpose of this study is to evaluate the uptake of ^68^Ga-DOTA-TATE in NB xenograft models and correlate this uptake with the expression levels of SSTR2 and, therefore, identify biomarkers which can predict the therapeutic effects of ^177^Lu-DOTA-TATE.

## 2. Materials and Methods

### 2.1. Materials and Reagents

Gallium-68 and lutetium-177 radiolabeled DOTA-TATE were supplied by the Centre for Probe Development and Commercialization (CPDC, Hamilton, ON, Canada). ^68^Ga-DOTA-TATE was produced with the specific activity of 41.2 ± 9.9 GBq/*μ*mol and with the radiochemical purity of >97% at all cases. NET antibody (NET17-1) was purchased from MAb Technologies (Stone Mountain, GA), and SSTR2 antibody (ab134152) was obtained from Abcam (Cambridge, MA). Triton X-100, ethylenediaminetetraacetic acid (EDTA), and sodium dodecyl sulfate (SDS) were purchased from Sigma Chemical Company (St Louis, MO).

### 2.2. Cells and Cell Culture

Eight NB cell lines (NUB-7, SK-N-BE(2), BE(2)C, LAN-5, SH-SY5Y, CHLA-15, CHLA-20, and CHLA-90) were selected to represent a panel of cell lines with different biological and genetic backgrounds of NB (Table 1). NUB-7, LAN-5, SK-N-BE(2), BE(2)C, and SH-SY5Y neuroblastoma cells were kindly provided by Dr. Herman Yeger (The Hospital for Sick Children, Toronto, ON, Canada). CHLA-15, CHLA-20, and CHLA-90 were obtained from the Children's Oncology Group Cell Culture and Xenograft Repository (http://www.cogcell.org/) under a signed and approved Material Transfer Agreement. Cell line authentication was performed using short tandem repeats (STR) DNA profiling (Promega's GenePrint® 10 System) [12] conducted by the Genetic Analysis Facility at the Centre for Applied Genomics of The Hospital for Sick Children. The DNA (STR) profile for all cell lines was matched to the profile listed in the Children's Oncology Group (COG) STR Database (http://strdb.cogcell.org/). CHLA-15, CHLA-20, and CHLA-90 neuroblastoma cells were cultured in Iscove's modified Dulbecco's medium supplemented with 3 mM L-glutamine, 5 *μ*g/mL insulin, 5 *μ*g/mL transferrin, and 5 ng/mL selenous acid (ITS Culture Supplement; Collaborative Biomedical Products, Bedford, MA) and 20% fetal bovine serum (FBS). NUB-7, LAN-5, SK-N-BE(2), BE(2)C, and SH-SY5Y neuroblastoma cells were cultured in *α*-MEM supplemented with 10% FBS.

### 2.3. RT-PCR

Total cellular RNA was prepared using Qiagen RNeasy mini kit (Qiagen, Valencia, CA) according to the manufacturers' instruction. Residual DNA was eliminated using the Qiagen RNase-Free DNase Set. cDNAs were synthesized from 2 *μ*g of RNA with the Superscript II™ Reverse Transcriptase (Invitrogen). PCR was performed using 1 *μ*L of cDNA in the PCR buffer supplemented with 0.2 mM of dNTP, 2.5 units of Taq polymerase (Biorad and ThermoFisher Scientific), and 0.5 *μ*M of each sense and antisense primer. The following primers were used: SSTR2 forward 5′-GGTGAAGTCCTCTGGAATCC-3′ and reverse 5′-CCATTGCCAGTAGACAGAGC-3′; NET forward 5′-CTCAAGGAGGCCACGGTATGGATCG-3′ and reverse 5′-ACCTGGAAGTCATCAGCCAGTCCGG-3′; GAPDH forward 5′-CTGTCCAGTTAATTTCTGACC-3′ and reverse 5′-CTTTGTACATGGTATTCACCAC-3′. PCR products were run on a 1.5% agarose (Invitrogen) with a 100 bp marker (Thermo Fisher Scientific) and stained with ethidium bromide. Gel pictures were taken using the AlphaImager™ 2200 (Alpha Innotech, Kasendorf, Germany).

### 2.4. Western Blot

The protein lysates were analyzed by Western blot for SSTR2 and norepinephrine transporter (NET). Briefly, cells were lysed in lysis buffer and denatured. Samples were separated using 10% Bis-Tris precast gels (Invitrogen), followed by transfer to a PDVF membrane. After blocking, all membranes were incubated overnight at 4°C in TBST (Tris-buffered saline, 0.1% Tween 20) buffer containing the primary antibodies. Primary antibody complexes were then detected using horseradish peroxidase- (HRP-) conjugated secondary antibodies. Protein bands were revealed with SuperSignal™ West Pico Chemiluminescent Substrate (Thermo Fisher Scientific). NET protein expression in CHLA-15, SK-N-BE(2), and BE(2)C xenografts was quantified densitometrically using ImageJ software (NIH, USA) and normalized with respect to the corresponding expression of *β*-actin.

### 2.5. Tissue Preparation for Western Blot

Xenograft tumors were snap frozen in liquid nitrogen immediately after harvesting and stored at −80°C until ready for processing. Tumor tissue samples were homogenized in a RIPA buffer (150 mM NaCl, 50 mM Tris-HCl, 0.5 mM EDTA, 1% Triton X-100, and 0.1% SDS) plus complete protease inhibitor cocktail (Complete Protease Inhibitor Tablets; Boehringer Mannheim, Ingelheim am Rhein, Germany). Homogenates were then centrifuged at 100,000 ×g for 45 minutes at 4°C. The supernatants were assayed for protein content, aliquoted, and stored at −80°C. 25 *μ*g of lysate was subjected to future protein analysis by SDS-PAGE and Western blot analysis.

### 2.6. Mouse Xenograft Models

All animal studies were approved by Animal Care Committee at the Hospital for Sick Children and at the University Health Network (Toronto, ON, Canada). Four- to 6-week-old, female, nonobese diabetic, severe combined immunodeficiency (NOD/SCID) mice were purchased from Jackson Laboratory (Bar Harbor, ME). CHLA-15, SK-N-BE(2) and BE(2)C cells were used to establish murine models. Briefly, tumor cells were washed three times with Hanks' Balanced Salt Solution (HBSS) before injection. Cell suspensions were mixed 1 : 1 with Growth Factor Reduced Matrigel Matrix (BD Bioscience, Mississauga, ON, Canada). Subcutaneous xenografts were developed by injecting 1 × 10^6^ tumor cells subcutaneously into the dorsal upper flank of NOD/SCID mice.

### 2.7. Micro-PET/CT Mouse Imaging

Animals were prepared for imaging such that when the tumor xenografts reach a diameter of approximately 1 cm, the mice were anesthetized with 2% isoflurane in the medical air (1.0 L/min) and injected intravenously (IV) via the tail vein [13] with 11.7 MBq ± 2.5 MBq of ^68^Ga-DOTA-TATE. Images were acquired on a Focus 220 micro-PET scanner (Siemens Preclinical Solutions, Knoxville, TN) at 1 hour after injection using a Minerve imaging bed (Esternay, France) to maintain body temperature at 37°C. Images were reconstructed in a 256 × 256 matrix and a zoom of 6.5 using an ordered subset expectation maximization (OSEM), followed by a maximum a posteriori probability reconstruction algorithm with no attenuation correction. Quantification was performed by volume-of-interest (VOI) analysis using Inveon Research Workplace (IRW) software (Siemens). Tumor volume was obtained by summing multiple 2-dimensional regions of interest from consecutive tomographic planes encompassing the entire tumor volume on fused PET-CT slices. Tumor uptake was expressed as the mean ± SD percentage injected dose per gram (%ID/g).

Immediately after small-animal PET, the Minerve imaging bed with the mouse was transferred to an eXplore Locus Ultra Preclinical CT scanner (GE Healthcare, London, ON, Canada). The micro-CT scan of the mouse was acquired with routine acquisition parameters (80 kV, 70 mA, 16 sec per rotation). PET and CT images were coregistered using IRW software. The micro-CT scan was used for anatomic referencing and for delineating the aforementioned VOIs.

To verify the accuracy of %ID values, a 10 mL specimen of ^68^Ga with known radioactivity was scanned on the micro-PET. The image-derived concentration was compared with the concentration calculated from the same radioactivity measured by the radioisotope dose calibrator (Model CRC® -15R, Capintec Inc., Ramsay, NJ). The difference in the two concentrations was less than 10%.

### 2.8. ^123^I-MIBG SPECT/CT Imaging

1 × 10^6^ NB tumor cells were injected subcutaneously into the shoulder area of NOD/SCID mice. When tumor xenografts grew to an approximate diameter of 1 cm or more, each mouse was injected with 17.5 MBq ± 2.3 MBq of ^123^I-MIBG into a lateral tail vein. Five to six hours after injection, both CT and SPECT imaging were performed using a preclinical nanoSPECT/CT system (Bioscan, Washington, DC). For imaging, mice were anesthetized with 1.5% isoflurane and medical air at 1.0 L/min. For anatomical reference, the cone-beam CT scan was acquired first at 45 kVp and 177 *µ*A. Image slices were reconstructed in a 176 × 176 matrix with a fast filtered back-projection algorithm using InVivoScope® 1.43 software (Bioscan, USA).

For the SPECT scan, a 20% window was set around the 159 keV principal gamma-photon of I-123. In this multiplexed multipinhole SPECT system, the 1.4 mm nine-pinhole mouse “standard” collimators were attached to each of the four detector heads consisting of NaI(Tl) crystals. Photons were acquired for about 150 s/projection and 24 projections per detector head in a 256 × 256 matrix for a total imaging time ranging from 60 to 75 minutes. SPECT data were reconstructed by ordered subset expectation maximization (OSEM) methods with four subsets of data undergoing 9 iterations each using InVivoScope® 1.43. The CT and SPECT slices were then coregistered. Image analysis and volume-of-interest (VOI) quantification were performed using VivoQuant® 2.5 (Mediso/inviCRO, Boston, MA). The activity concentration in the VOI for the whole tumor was divided by the activity concentration in the VOI for the hind limb muscle in order to calculate the tumor-to-muscle ratio (*T*/*M*).

### 2.9. Autoradiography

After the ^68^Ga-DOTA-TATE PET/CT and ^123^I-MIBG SPECT/CT studies, CHLA-15, SK-N-BE(2), and BE(2)C xenografts were harvested, cut in half, embedded in Tissue-Tek® optimum cutting temperature (OCT) compound (Tissue-Tek, Sakura, Torrance, CA), along with a piece of forelimb muscle as control, and frozen on liquid nitrogen vapor. Frozen blocks were transferred to the STTARR correlative pathology lab on dry ice, and serial frozen sections of alternating 5 *µ*m (for immunohistochemistry) and 50 *µ*m thickness (for autoradiography) were cut, placed on glass slides, and left to dry for 20 minutes. After sections were completely dry, the 50 *µ*m sections were placed in a custom-built 16-slide holder that held the frozen sections in close proximity to a storage phosphor screen (Cyclone Plus Storage Phosphor System, Perkin Elmer, Shelton, CT, USA), with a layer of plastic wrap separating them. In some instances, a piece of filter paper with serial dilutions of radiotracer was included in the cassette for use as a standard and to check the linearity of the film. This cassette was maintained at −20°C for a period of time equating to 10 half-lives of activity. The timing of tumor resection and contact with phosphor screen were recorded for all cases. Following completion of 10 half-lives of decay, the phosphor screen was removed from the cassette and developed on the Cyclone Plus imaging system at 600 dpi resolution, with a written record of slide locations on the screen. Image quantification was performed in ImageJ software, in which regions of interest (ROIs) were drawn around each tumor and corresponding piece of muscle tissue, and mean phosphor intensity was recorded in each region. A ROI corresponding to background signal was also recorded, taken from the region of screen not containing any slides or tissue. Mean tumor-to-muscle ratios were calculated by division of mean per-pixel intensity in tumor (subtracting background) divided by mean per-pixel muscle intensity, subtracting background.

### 2.10. SSTR2 Immunohistochemistry

SSTR2 immunofluorescence staining was performed on 5 *μ*m thick frozen sections. Tissue sections were fixed for 10 minutes in acetone and allowed to air dry for 5 minutes. Endogenous biotin, biotin receptors, and avidin sites were blocked with the Avidin/Biotin Blocking Kit (SP-2001, Vector Laboratories, Burlingame, CA). The tissue sections were incubated with rabbit anti-SSTR2 antibody (1 : 100; ab9550, Abcam) for 1 hour at room temperature. Detection of the rabbit antibodies was performed by incubation with Texas Red goat anti-rabbit IgG antibody (1 : 200; TI-1000, Vector Laboratories) for 30 minutes. For the positive control of SSTR immunohistochemistry, normal pancreas islets were used. Negative controls were done by omitting the specific primary antibodies and processed in the same way. The tissue sections were washed and then mounted with Vectashield mounting medium with DAPI (H-1200, Vector Laboratories). Whole-slide scanned immunofluorescence images were acquired on a TissueScope 4000 (Huron Technologies, Waterloo ON, Canada) at 1 *µ*m/pixel resolution. Coregistration of autoradiography with immunofluorescence was performed by upsampling the autoradiography image (600 dpi, which equates to 42 *µ*m/pixel resolution) of the 50 *µ*m adjacent section to match the immunofluorescence image (imaged at 1 *µ*m/pixel resolution), followed by rigid registration using a customized MATLAB script.

### 2.11. *In Vivo* Treatment with ^177^Lu-DOTA-TATE

Drug treatment commenced when the tumor sizes reached 0.5 cm in diameter. Animals were randomized into two groups, each with 7 animals: the control group and the ^177^Lu-DOTA-TATE treatment group. A single dose of ^177^Lu-DOTA-TATE (20 MBq) [14] was administered as treatment. Control mice received the same volume of saline. Tumor growth was monitored by measuring tumor dimensions using a digital caliper. Tumor volume was calculated as width^2^ × length × 0.5. When tumor volume reached 3 cm^3^, mice were sacrificed, and tumors were dissected and weighed. Tumor growth curves consisting of the tumor volumes at different time points were plotted. During the study, the mice were observed daily for possible adverse effects due to treatments. Morbidity signs of ill health such as ruffled/thinning fur, abnormal behaviors, or local erosion from the tumor, were observed. Animal body weight was also monitored for general toxicity.

### 2.12. Statistical Analysis

Data from different experiments were presented as mean ± SD. Two-tailed, unpaired Student's *t*-tests were performed to compare the uptake values obtained from SPECT/CT imaging and tumor growth in two different groups. *T*/*M* ratios of ^123^I-MIBG uptake and of ^123^I-MIBG autoradiography in different groups were analyzed by one-way analysis of variance (ANOVA) and Tukey's test. Comparison of NET expression between different groups was analyzed by the nonparametric Kruskal-Wallis test with Dunn's multiple comparison tests. Statistical significance was achieved with a two-sided *P* < 0.05. All statistics were generated using GraphPad Prism software version 6.

## 3. Results

### 3.1. Variable Expression Levels of SSTR2 and NET in Neuroblastoma (NB) Tumor Cell Lines

**Figure 1:**
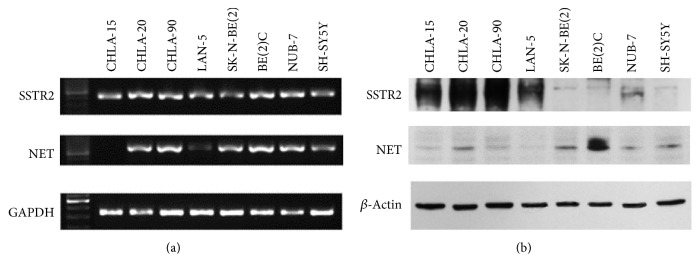
mRNA expression level and Western blotting analyses of SSTR2 and NET. (a) RNA was isolated from different neuroblastoma cell lines, converted into cDNA, followed by RT-PCR with SSTR2 and NET specific primers. The GAPDH gene was used as a reference gene. (b) Protein lysates were prepared from different neuroblastoma cell lines. Protein samples were separated by polyacrylamide gel electrophoresis. Expression of SSTR2 and NET proteins was visualized using specific antibodies. *β*-Actin was used as internal loading control.

The expression of somatostatin receptors (SSTR2) and NET in NB cell lines was determined using RT-PCR (Figure 1(a)) and Western blot (Figure 1(b)). Although different NB cell lines showed similar SSTR2 mRNA expression levels (Figure 1(a)), marked variation of SSTR2 protein expression was observed (Figure 1(b)). In some NB cell lines such as CHLA-15, CHLA-20, CHLA-90, and LAN-5, a prominent SSTR2 expression was detected, whereas, in others, a low level of SSTR2 expression was identified (Figure 1(b)). A similar variation was observed for the expression of NET, a primary transporter responsible for specific active cellular uptake of MIBG [15] (Figures 1(a) and 1(b)). Interestingly, some high SSTR2-expressing cell lines, CHLA-15, CHLA-90, and LAN-5, showed low expression levels of NET, which makes SSTR2 a potential alternative molecular target for NB imaging or treatment, especially for MIBG nonacid tumors.

In addition, SSTR2 or NET expression did not appear to be correlated with MYCN amplification or p53 mutation status. In some cell lines, two bands were detected using the monoclonal SSTR2 antibody (ab134152, Abcam), which is consistent with a previous report in which two bands were also detected in IMR-32 neuroblastoma cell lysates [16].

### 3.2. Uptake of ^68^Ga-DOTA-TATE Correlates with SSTRT2 Expression in NB Xenografts

**Figure 2:**
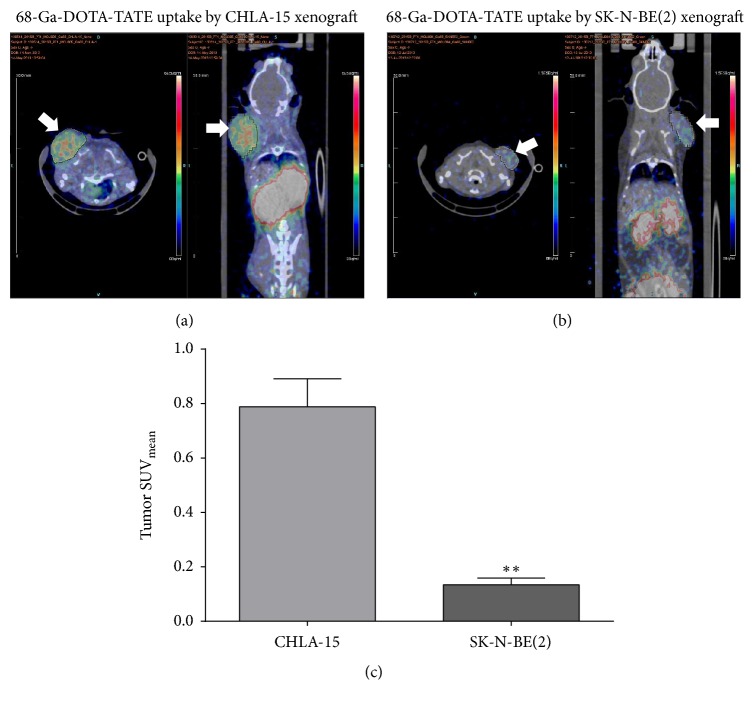
Representative micro-PET/CT images at 1 hour after injection of 10 MBq of  ^68^Ga-DOTA-TATE in the CHLA-15 (a) and SK-N-BE(2) (b) tumor-bearing NOD/SCID mice. Images were presented in the axial (left) and coronal (right) orientations. The white arrows denote localized tumor on the shoulder. (c) Standardized Uptake Values (SUV) in CHLA-15 and SK-N-BE(2) xenografts were calculated using the formula: SUV = *C*_PET_(*T*)/(Injected  dose/Bodyweight). The difference was significant between CHLA-5 and SK-N-BE(2) tumors (^*∗∗*^*P* < 0.01). Two-tailed unpaired *t*-tests were performed to compare the SUV_mean_ values obtained.

DOTA-TATE has a high affinity for SSTR2* in vitro*. In order to assess the relationship between ^68^Ga-DOTA-TATE uptake and SSTR2 expression, we selected a high SSTR2-expressing NB cell line, CHLA-15, and a low SSTR2-expressing cell line, SK-N-BE(2), for* in vivo* PET/CT tumor imaging. Tumor uptake was expressed as Standardized Uptake Value (SUV). As shown in Figures 2(a)–2(c), we observed a significant difference in the uptake of ^68^Ga-DOTA-TATE between CHLA-15 and SK-N-BE(2) xenografts. The mean tumor uptake value of ^68^Ga-DOTA-TATE was significantly higher in the CHLA-15 xenografts (0.79 ± 0.10% ID/g; *P* = 0.0003), compared to SK-N-BE(2) tumors (0.13 ± 0.02% ID/g; *P* < 0.01).

### 3.3. Histological Colocalization of SSTR2 and ^68^Ga-DOTA-TATE Uptake

**Figure 3:**
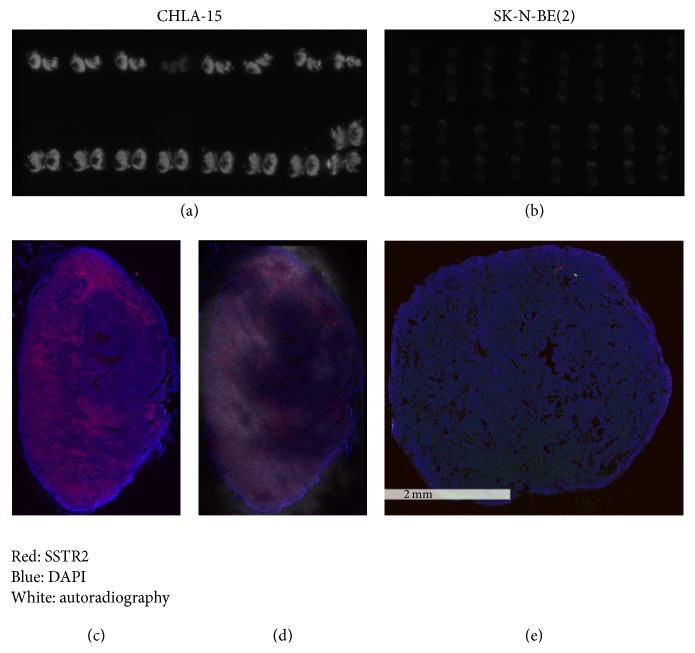
Colocalization of SSTR2 and autoradiography on CHLA-15 xenografts. CHLA-15 and SK-N-BE(2) tumors were removed immediately after PET/CT imaging. The spatial distribution of the ^68^Ga-DOTA-TATE uptake was visualized by autoradiography (white signal) in the serial sections of CHLA-15 (a) and SK-N-BE(2) (b) tumors. (c) Representative CHLA-15 tumor section was stained for SSTR2 (red fluorescence) and DAPI (blue fluorescence). (d) The merging image of SSTR2 immunostaining and autoradiograph of the same CHLA-15 tumor section. (e) A representative SK-N-BE(2) tumor section stained for SSTR2 (red fluorescence) and DAPI (blue fluorescence).

To further evaluate the relationship between SSTR2 expression and ^68^Ga-DOTA-TATE uptake, we performed SSTR2 immunostaining and* ex vivo* autoradiography with CHLA-15 and SK-N-BE(2) xenograft sections. Consistent with the PET results, CHLA-15 tumors showed significantly higher accumulation of ^68^Ga-DOTA-TATE as compared to SK-N-BE(2) tumors (Figures 3(a) and 3(b)). We also observed spatial heterogeneity for both SSTR2 expression (Figure 3(c)) and ^68^Ga-DOTA-TATE accumulation (Figures 3(a) and 3(d)). When we merged the SSTR2 fluorescent staining and autoradiography images, we observed intratumoral colocalization of SSTR2 expression and ^68^Ga-DOTA-TATE uptake (Figure 3(d)). Tumor regions with a high number of SSTR2-positive cells corresponded to focal areas of increased radioactivity. In SK-N-BE(2) tumors, both SSTR2 expression and ^68^Ga-DOTA-TATE autoradiography signals were too weak to be detected (Figure 3(e)).

### 3.4. Therapeutic Effects of ^177^Lu-DOTA-TATE on the CHLA-15 Xenograft Model

**Figure 4:**
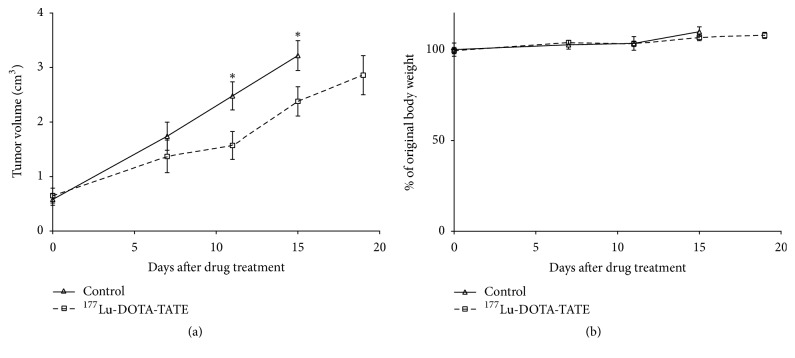
Antitumoral effects of ^177^Lu-DOTA-TATE in the CHLA-15 neuroblastoma model. (a) Mice (*n* = 7) with subcutaneous CHLA-15 xenografts were treated with one dose of 20 MBq of ^177^Lu-DOTA-TATE. Control mice (*n* = 7) received saline. Tumor volume was measured and plotted as shown. Values are stated as mean ± SE; ^*∗*^*P* < 0.05. (b) Animal body weight was monitored in CHLA-15 tumor-bearing mice with/without ^177^Lu-DOTA-TATE treatment.

To verify the therapeutic effects of targeting SSTR2 with ^177^Lu-DOTA-TATE, we treated CHLA-15 tumor-bearing mice with ^177^Lu-DOTA-TATE at the dose of 20 MBq/animal. After 12 days, we started to observe significant tumor growth inhibition in the CHLA-15 xenograft model compared to control tumors (*P* < 0.05) (Figure 4(a)). From the slope of tumor growth curve, Lu-177-DOTA-TATE treated tumors regained tumor growth rate after day 12, which indicates that single dose Lu-177-DOTA-TATE may not achieve long-lasting antitumor effects. No overall toxicity with respect to body weight loss was observed at the dose of 20 MBq/animal (Figure 4(b)).

### 3.5. ^123^I-MIBG Uptake by NB Xenografts Not Related to Their NET Expression* In Vitro*

**Figure 5:**
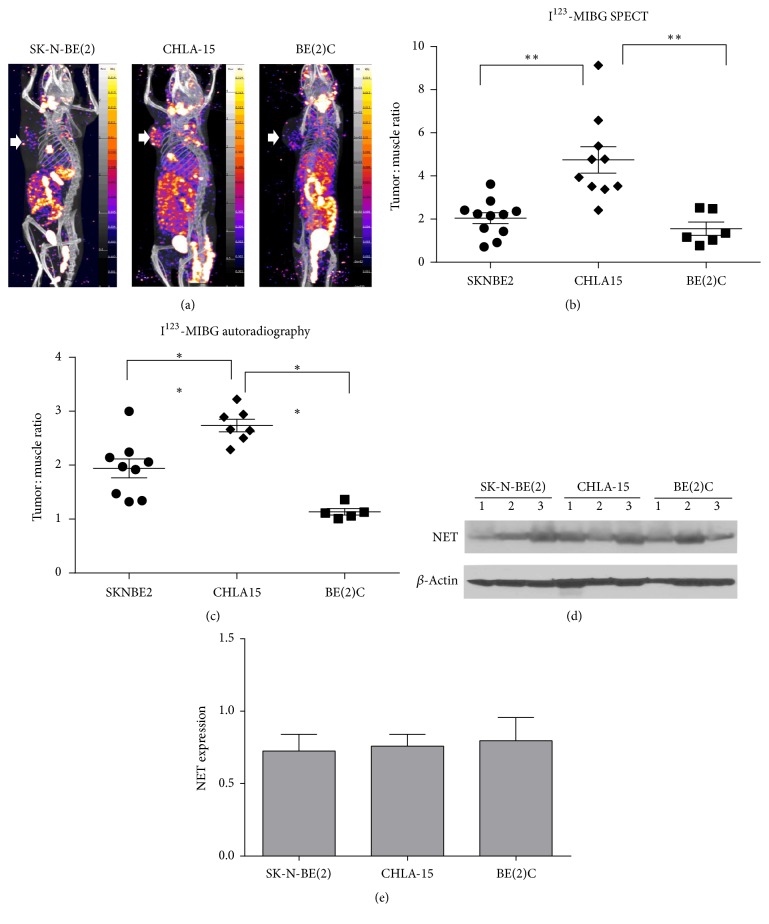
(a) Example images of ^123^I-MIBG SPECT/CT in the SK-N-BE(2), CHLA-15, and BE(2)C xenograft models. The white arrows denote localized tumor on the shoulder. (b) Scatter plots of the tumor-to-muscle (*T*/*M*) ratios of ^123^I-MIBG uptake in the SK-N-BE(2), CHLA-15, and BE(2)C xenograft models derived from SPECT/CT imaging. One-way analysis of variance (ANOVA) with Tukey's test was used for statistical analysis. ^*∗*^*P*<0.05; ^*∗∗*^*P* < 0.01. (c) Scatter plots of the tumor-to-muscle (*T*/*M*) ratio of ^123^I-MIBG autoradiography in the SK-N-BE(2), CHLA-15, and BE(2)C xenograft models. One-way ANOVA with Tukey's test was used for statistical analysis. ^*∗*^*P* < 0.05; ^*∗∗*^*P*<0.01. (d) Western Blot of NET with homogenized CHLA-15, SK-N-BE(2), and BE(2)C xenografts. Three tumors were randomly selected from three groups for Western blot analysis. (e) Quantitative analysis of NET protein expression in CHLA-15, SK-N-BE(2), and BE(2)C xenografts. NET protein expression in Western blot images was quantified densitometrically using ImageJ software (NIH, USA) and normalized with respect to the corresponding expression of *β*-actin. Comparison of NET expression between different groups was analyzed by the nonparametric Kruskal-Wallis analysis with Dunn's multiple comparison tests. No significant difference of NET expression was observed between CHLA-15, SK-N-BE(2), and BE(2)C xenografts.

To compare the effectiveness between ^68^Ga-DOTA-TATE PET and ^123^I-MIBG SPECT in our preclinical models, we further assessed ^123^I-MIBG uptake in three neuroblastoma xenograft models, including NB cells expressed high, intermediate, and low amount of SSTR2 protein* in vitro*, BE(2)C, SK-N-BE(2), and CHLA-15 cells, respectively. As shown by ^123^I-MIBG SPECT (Figures 5(a) and 5(b)) and ^123^I-MIBG autoradiography (Figure 5(c)), uneven marginal uptake of ^123^I-MIBG was observed in all three BE(2)C, SK-N-BE(2), and CHLA-15 xenograft tumors. Also,* in vivo  *^123^I-MIBG uptake is not related to* in vitro* NET expression levels in three selected NB cell lines. Although BE(2)C cells had a relatively higher NET expression* in vitro*, their* in vivo  *^123^I-MIBG uptake is at the similar level as the background, with the *T*/*M* ratio of 1.13 ± 0.13. We were not able to detect positive signals from NET immunohistochemistry staining with harvested NB xenografts. In order to compare the NET expression* in vivo*, we ran a Western blot of NET with homogenized tumor samples (Figure 5(d)). We did not, however, observe a significant difference of NET expression between CHLA-15, SK-N-BE(2), and BE(2)C xenografts, which indicates a possible change of NET expression profile due to* in vitro* cell culture (Figure 5(e)).

## 4. Discussion

Neuroblastoma is an extremely heterogeneous disease, both biologically and clinically, comprising tumor cells with very different molecular features. Somatostatin receptors (SSTRs) are variably expressed in neuroblastoma cell lines and tumors, as demonstrated by autoradiography, Western blot, immunohistochemistry, and RT-PCR techniques [17–19]. In this study, we selected a panel of NBL cells lines with different biological and genetic backgrounds (Table 1). We detected SSTR2 mRNA in most of the selected cell lines, but only 4 out of 8 cell lines express high levels of SSTR2 protein, including CHLA-15, CHLA-20, CHLA-90, and LAN-5 cells. In addition, expression of SSTR2 was not related to the p53 mutation and MYCN amplification status. Since the commercially available NB cell lines are mostly derived from stage IV patient samples, we are currently conducting a large-scale Children's Oncology Group biological study (COG ANBL14B3) with both high-risk and non-high-risk NB patient specimens to investigate the relationship between SSTR2 expression and clinical features.

Peptide-receptor imaging and therapy with radiolabeled somatostatin analogs are an established and effective treatment option for adult patients with SSTR-positive neuroendocrine tumors [4]. ^111^In-[diethylenetriaminepentaacetic acid (DTPA)]octreotide (Octreoscan; Mallinckrodt), a somatostatin analog, has been used for more than 15 years in the diagnosis and staging of SSTR-positive tumors. Nevertheless, it has a restricted ability to identify lesions smaller than 1 cm and to obtain a good spatial resolution, even when using SPECT rather than planar imaging [20]. In recent years, new PET-based radiopharmaceuticals targeting somatostatin receptors have been developed to address these issues. Several studies have demonstrated that ^68^Ga-labeled DOTA-TOC or DOTA-TATE PET combined with CT has distinctly higher sensitivity and improved spatial resolution for the detection of SSTR-positive neuroendocrine tumors compared to scintigraphy with conventional SPECT imaging using Octreoscan [4, 21]. In this study, we successfully imaged ^68^Ga-DOTA-TATE uptake in SSTR2-positive neuroblastoma xenografts with micro-PET/CT. We also observed intensive ^68^Ga-DOTA-TATE uptake in MIBG-low-avidity CHLA-15 xenografts.

^131^I-MIBG has been used over the past 15 years in multimodal therapy as a radiotherapeutic agent in relapsed and refractory NB patients [22]. However, only 30–40% of children with chemotherapy-refractory disease respond to ^131^I-MIBG, and the responses are usually only transient [22–24]. In this study, we observed low ^131^I-MIBG avidity in all selected NB cell lines. Although BE(2)C cells showed relatively elevated NET expression* in vitro*, we were not able to detect positive NET immunostaining with harvested BE(2)C xenografts. Western blot of NET with homogenized tumor samples showed similar NET expression levels between CHLA-15, SK-N-BE(2), and BE(2)C xenografts, which indicates a possible change of NET expression profile* in vivo*. We also demonstrated the complementary role of somatostatin receptor imaging in detecting additional sites of neuroendocrine tumors that were not visualized with ^123^I-MIBG scintigraphy. Several clinical studies also confirmed discordant uptake patterns of MIBG and somatostatin receptor expression in some NB tumors [25]. Kroiss et al. [26], for example, described that somatostatin receptor imaging with ^68^Ga-DOTA-TOC PET was able to detect the sites of disease in 2/4 patients with NB which were not visible by ^123^I-MIBG. Numerous other studies in patients with neuroendocrine tumors have similarly demonstrated the complementary role of somatostatin receptor imaging in detecting additional sites of disease that were not visualized with ^123^I-MIBG scintigraphy [27]. In this study, we observed discordant expression of NET and SSTR2 expression in NB-cell lines. CHLA-15 has a lower level of NET expression but higher SSTR2 expression compared to SK-N-BE(2) cells.* In vivo* PET/CT imaging demonstrated high ^68^Ga-DOTA-TATE uptake in CHLA-15 xenografts compared to SK-N-BE(2) tumors. Consequently, somatostatin receptor expression and DOTA-TATE imaging could serve as an adjunct to existing diagnostic and therapeutic methods. They may provide valuable information for the pretherapeutic staging of the disease and impact patient outcomes.

One of the major goals of our study is to understand how the molecular expression of SSTR2 determined the responses of radioactively labeled DOTA-TATE. DOTA-TATE can also be radiolabeled with ^177^Lu for targeted *β*^−^-particle radiotherapy. ^177^Lu has a similar physical half-life as ^131^I. ^177^Lu emits a medium-energy *β*^−^-particle resulting in localized energy deposition; thus the targeted tissue receives low-dose-rate radiation exposure. Mouse studies of solid tumor xenografts found ^177^Lu to be superior to other radiolanthanides in effecting tumor control with pretargeted therapy [28]. We demonstrated the antitumor effect of ^177^Lu-DOTA-TATE on CHLA-15 xenografts which have high SSTR2 expression and elevated uptake of ^68^Ga-DOTA-TATE. These characteristics may be valuable in future clinical trials: both ^68^Ga-DOTA-TATE PET imaging and SSTR2 protein profile could serve as indicators for ^177^Lu-DOTA-TATE treatment response. This avenue needs to be evaluated further in clinical scenarios.

## 5. Conclusions

This study has allowed us to demonstrate the association between SSTR2 expression and ^68^Ga-DOTA-TATE uptake, which potentially leads to the antitumor activity of ^177^Lu-DOTA-TATE in NB preclinical models. Histological colocalization of SSTR2 and ^68^Ga-DOTA-TATE was also observed in our study. SSTR2 expression therefore could be used as a potential biomarker for predicting drug response to ^177^Lu-DOTA-TATE radiotherapy. Moreover, in our model, we demonstrated that ^68^Ga-DOTA-TATE PET is superior to ^123^I-MIBG SPECT imaging in detecting NB xenograft tumors. The absence of significant difference of NET expression between various NB xenografts models and discordant* in vitro* and* in vivo* NET expression represent a limitation of our study which will require further investigation.

The ongoing COG study looking at the prevalence of SSTR2/NET expression in high-risk NB patients may allow us to identify a subset of patients who could benefit from this new SSRT2 targeted radiotherapeutic modality, in particular for the small number of patients demonstrating MIBG-nonavid tumors.

## Figures and Tables

**Table 1:** Neuroblastoma cell lines used in this study.

Cell line	Site	Stage	Patient age	Phase of therapy	MYCN amp	p53 mutant
CHLA-15	Tumor	4	>1	DX	N	WT
CHLA-20	Tumor	4	1.5	PD-Ind	N	WT
CHLA-90	BM	4	8.5	PD-Auto-BMT	N	Mut
LAN-5	BM	Unknown	0.4	Unknown	A	WT
NUB-7	LN	4s/4	0.7	Unknown	A	WT
SH-SY5Y	BM	4	4	PD-Ind	N	WT
BE(2)C	BM	4	2.2	PD-Ind	A	Mut
SK-N-BE(2)	BM	4	2.2	PD-Ind	A	Mut

BM = bone marrow; B = bone; L = liver; P = pulmonary; LN = lymph node; DX = at diagnosis; PD-Ind = progressive disease on induction chemotherapy; BMT = bone marrow transplantation; PD-Auto-BMT = relapsed after myeloablative chemo-radiotherapy followed by bone marrow transplantation; WT =wild type; Mut = mutant; N = nonamplified; A = amplified.
